# A molecular basis of human T cell receptor autoreactivity toward self-phospholipids

**DOI:** 10.1126/sciimmunol.aao1384

**Published:** 2017-10-20

**Authors:** Adam Shahine, Ildiko Van Rhijn, Tan-Yun Cheng, Sarah Iwany, Stephanie Gras, D. Branch Moody, Jamie Rossjohn

**Affiliations:** 1 Infection and Immunity Program and Department of Biochemistry and Molecular Biology, Biomedicine Discovery Institute, Monash University, Clayton, Victoria 3800, Australia.; 2 Australian Research Council Centre of Excellence in Advanced Molecular Imaging, Monash University, Clayton, Victoria 3800, Australia.; 3 Division of Rheumatology, Immunology and Allergy, Brigham and Women’s Hospital and Harvard Medical School, Boston, MA 02115, USA.; 4 Department of Infectious Diseases and Immunology, Faculty of Veterinary Medicine, Utrecht University, Yalelaan 1, 3584CL Utrecht, Netherlands.; 5 Institute of Infection and Immunity, Cardiff University School of Medicine, Heath Park, Cardiff CF14 4XN, UK.

## Abstract

Human T cell autoreactivity toward lipid antigens presented by CD1 proteins can manifest in numerous diseases, including psoriasis, contact hypersensitivities, and allergies. However, the molecular mechanisms for regulating T cell autoreactivity toward lipid antigens remain unclear. We determined the basis for T cell receptor (TCR) autoreactivity toward CD1b bound to self-phospholipids. The spectrum of self-antigens captured by CD1b skews toward abundant membrane phospholipids such as phosphatidylcholine and phosphatidylethanolamine. However, TCRs can specifically recognize rare phospholipids, including phosphatidylglycerol (PG). The structure of an autoreactive TCR bound to CD1b-PG shows that discrimination occurs through a marked induced fit movement of PG so that its polar head group fits snugly into the cationic cup of the TCR. Conversely, TCR binding toward ubiquitous self-phospholipids was sterically or electrostatically repelled. Accordingly, we describe a mechanism of TCR autoreactivity toward rare phospholipids and avoidance of autoreactivity to the most abundant self-phospholipids.

## INTRODUCTION

T cells, via their αβ T cell antigen receptors (TCRs), play a crucial role in protective immunity by specifically recognizing foreign peptide antigens when presented by molecules encoded by the major histocompatibility complex (MHC) (1). Precise specificity for the peptide, combined with the processes of thymic education and peripheral tolerance, allows MHC-reactive T cells to minimize reactivity toward self-peptide antigens (2), and in so doing limits autoimmune disease. Although we have a broad understanding of the molecular mechanisms limiting the MHC-mediated response to self-peptides (3), the basis of avoidance of self-reactivity toward lipidic autoantigens presented by the CD1 family is less clear.

The human CD1 family of antigen-presenting molecules (CD1a, CD1b, CD1c, and CD1d) is structurally related to MHC class I (MHC-I) molecules with the key exception that CD1 proteins bind lipidic antigens (4, 5). Each of the four types of human CD1 antigen-presenting molecules has a distinctly shaped antigen-binding cleft, differing subcellular routes of trafficking, and distinct modes of transcriptional regulation (4), suggesting that CD1 family members have distinct functions (6). Most studies on CD1 have focused on CD1d-restricted natural killer T (NKT) cells. Knowledge of CD1a-, CD1b-, and CD1c-restricted T cell responses, which are conserved in most mammals but are not found in mice, remains germinal (4).

CD1b is distinguished from other CD1 antigen-presenting molecules in several key features of its structure and function. CD1b proteins are inducibly expressed on macrophages and myeloid dendritic cells (DCs) in response to bacterial factors, Toll-like receptors, and interleukin-1β (IL-1β) (7). Among human CD1 proteins, only CD1b proteins have sequences in their cytoplasmic tails that bind to adaptor protein complex 3, which enable strong localization to lysosomes, where specialized antigen capture mechanisms occur in a low-pH environment (8, 9). The CD1b cleft is much larger than the corresponding clefts of other CD1 proteins (10–14). The unusually spacious cleft allows CD1b to capture either two small lipids or particularly large lipid antigens (15–17).

Although most studies of CD1b focus on infection (18), recent studies have identified a role for CD1b in T cell autoreactivity, including CD1 tetramer–guided studies in humans and human TCR transgenic mice (19–21), identifying self-phospholipids as antigenic targets. The human T cell response was triggered by phosphatidylglycerol (PG) (19), whereas structurally related phosphatidic acid (PA)–containing lipids mediated skin disease in mice (21). These CD1b-based studies find resonance in the CD1d and CD1a systems, because some NKT cells and other T cells recognize PG, phosphatidylethanolamine (PE), or phosphatidylcholine (PC) (22–24). Whereas anti-phospholipid antibodies have been studied as causes of thrombosis in autoimmune disease for decades (25), cellular phospholipids were generally thought to be inert for the activation of T cells. Therefore, a new perspective to arise from these studies is that PG, PC, PE, and related membrane phospholipids can be natural antigens for T cells. However, there is no overarching understanding of how T cells could discriminate among classes of cellular phospholipids or how abundant self-phospholipids could avoid generating T cell autoimmunity. Here, we use mass spectrometry and structural biology to determine how abundant phospholipid classes can be ignored, leading to specific recognition of rare self-phospholipid antigens.

## RESULTS

### PG is of low abundance in cells

**Fig. 1.**
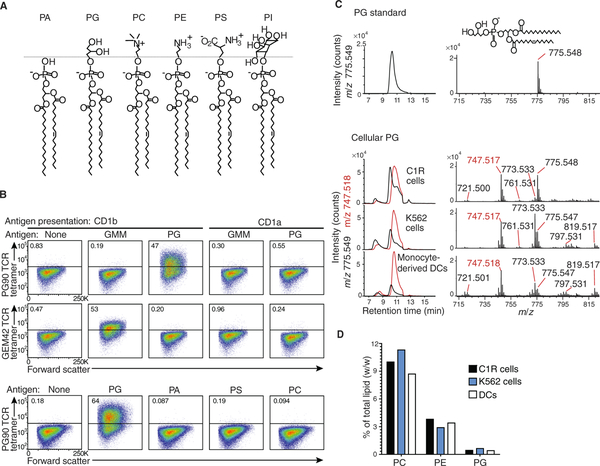
TCRs bind to CD1b-PG complexes formed in cells. (**A**) Cellular phospholipids share a PA core structure. PG, PC, PE, PS, and PI are distinguished by head groups above the dotted line. (**B**) C1R cells transfected with CD1a or CD1b were pulsed with the indicated lipid and stained with PG90 or GEM42 TCR tetramers. Gating strategy is shown in fig. S2. (**C**) Total cellular lipids were extracted from human monocyte-derived DCs, C1R cells, and K562 cells, separated using normal-phase HPLC, and detected as ion chromatograms that match the mass values for PG (*m/z* 775.5 and 747.5), which were previously established from TLC-MS. (**D**) MS signals for the abundant species of PG, PE, and PC. Experiments in (B) and (C) were performed at least twice, with similar results. Measurements in (D) were performed once in each of the three cell types.

Most membrane phospholipids have a PA core, with key subclasses defined by varying head groups attached to the phosphate moiety, including PE, PC, phosphatidylinositol (PI), phosphatidylserine (PS), and PG (Fig. 1A). Recently cloned TCRs, termed PG10 and PG90, mediate autoreactivity to target cells that express CD1b, and the response was augmented by adding micromolar quantities of PG (19). Thus, PG might function as a lipid autoantigen. However, CD1b-PG complexes were not directly detected, and PG might have acted through direct effects on T cells or membranes. To evaluate this hypothesis, we took two experimental approaches: generation of PG90 TCR tetramers and development of methods to detect CD1b-PG complex formation in cells.

First, we expressed the PG90 TCR and a CD1b–glucose monomycolate (GMM)–specific TCR termed GEM42 as control, containing a BirA tag that enabled binding to avidin (fig. S1) (26). In flow cytometric studies of a human B cell lymphoblastoid line, C1R, which does or does not express CD1b (C1R.CD1b and C1R.CD1a), the PG90 TCR tetramer showed trace (0.83%) staining of C1R.CD1b cells and bright staining after treatment of antigen-presenting cells (APCs) with PG (Fig. 1B and fig. S2). Conversely, no staining was seen for PG-treated C1R. CD1a or with GEM42 TCR tetramers on PG-treated C1R.CD1b cells, ruling out the nonspecific adherence of TCR tetramers to cells. Moreover, low or absent PG90 TCR tetramer staining was observed when C1R.CD1b cells were treated with other self-phospholipids, including PS, PA, and PC (Fig. 1B). CD1b tetramers loaded with PS and PA bound the PG90 TCR in a previous study (19). The differential ability of CD1b tetramers and TCR tetramers to bind to their TCR and CD1b-phospholipid targets, respectively, is likely attributable to the differing thresholds of binding. Overall, the data demonstrate that PG is efficiently captured by cellular CD1b proteins, forming CD1b-PG complexes that specifically bind to the PG90 TCR.

The low absolute staining of untreated C1R.CD1b (Fig. 1B) and relatively low cytokine secretion by autoreactive T cells (19) suggest that APCs have some mechanism for limiting autoreactivity to self–CD1b-PG complexes. One simple, “rare antigen hypothesis” is that PG is normally expressed at low absolute concentrations and, unlike abundant membrane lipids, is somewhat specifically expressed in mitochondria (27). Previous studies suggest that PG is less than 1% of lipid in mouse hepatocytes (28). Initial thin-layer chromatography–mass spectrometry (TLC-MS) studies of K562 and C1R cells detected a weakly staining spot that nearly comigrated with authentic PG (fig. S4) and identified key PG species with overall lipid lengths of C34:1 [mass/charge ratio (*m/z*) 747.4] or C36:1 (*m/z* 775.5). However, sulfatides and other comigrating lipids prevented quantitation (fig. S3). Next, we validated a system of high-performance liquid chromatography (HPLC) with sensitive quadrupole–time-of-flight (QToF) MS detection. HPLC rendered pure cellular PG at ~11 min (Fig. 1C), which showed the expected peak heterogeneity from naturally occurring isobars. After generating standard curves (fig. S5), we extended the analysis to common membrane lipids and to human monocyte-derived DCs as an example of a primary CD1b^+^ APCs. PG comprised ~1% of cellular lipid in all cell types, which is ~10- to 20-fold lower than measured PE and PC concentrations (Fig. 1D).

### CD1b-PG complex formation in cells

**Fig. 2.**
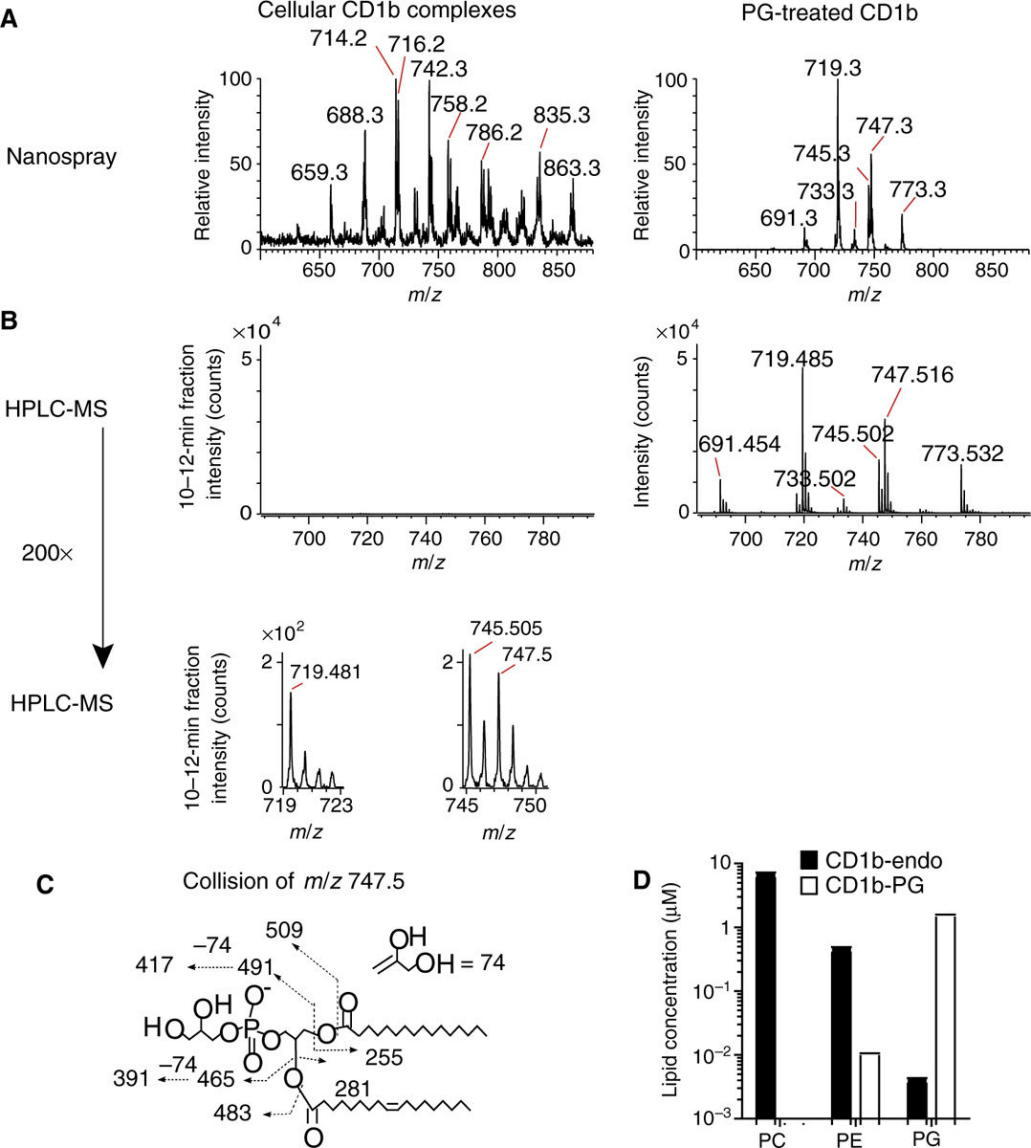
CD1b lipid complexes formed in cells. (**A**) CD1b protein complexes containing endogenous lipids (CD1b-endo) were extracted and analyzed using negative mode shotgun nanoelectrospray ionization ion trapping MS (nanospray). (**B**) Proteins prepared as in (A) were also analyzed by normal-phase HPLC-MS using QToF detection (HPLC-MS) measuring ions nearly coeluting with a PG standard at ~11-min retention time. The bottom panel shows signals magnified by 200-fold. (**C**) The identity of the compound as PG was established on the basis of its mass (*m/z* 747.511) and collision-induced dissociation MS. (**D**) Absolute lipid concentrations of cellular (CD1b-endo) and PG-treated proteins (CD1b-PG) were determined using authentic external standards (fig. S5).

Next, we sought to directly isolate CD1b-PG complexes and compared them with the broader spectrum of CD1b-lipid complexes formed. We expressed transmembrane-truncated CD1b–β_2_ microglobulin (β2m) complexes in human embryonic kidney (HEK) 293S cells, which were subjected to elution of lipids. Nano–electrospray ionization (ESI)–MS of lipids eluting from CD1b, but not from HLA-DR4-CLIP, generated rich spectra (Fig. 2A), indicating that the lipids were likely derived from the hydrophobic cleft of CD1b (fig. S6). We observed many ions in the mass range (*m/z* 600 to 900) typical for CD1 ligands, but no ions matching the mass of PG. In contrast, in vitro exposure of CD1b proteins to PG, followed by washing and lipid elution, generated a mass spectrum dominated by PG signals (*m/z* 719.3, 747.3, and 773.3) (Fig. 2A and fig. S6), demonstrating efficient PG loading of CD1b proteins and sensitive detection of PG.

After nanospray failed to detect PG, we applied more sensitive QToF methods, focusing specifically on PG coeluting lipids in the ~11-min retention time window. This approach demonstrated strong PG signals in the range of ~10^4^ counts in eluents of PG-treated CD1b, but not in cell-derived CD1b eluents (Fig. 2B). However, after increasing the gain 200-fold, we detected weak ions matching the mass (Fig. 2B) and collisional patterns (Fig. 2C) of PG. These data supported the rare antigen hypothesis, demonstrating PG capture near the detection limit of a sensitive technique. Using standards to estimate absolute concentrations(fig. S5), CD1b acquired PG in cells at 100- to 5000-fold lower concentrations than PC and PE. Again, loading controls (Fig. 2D) showed that this low rate of PG acquisition does not result from an intrinsic inability of CD1b to capture PG and more likely results from its scarcity or infrequent contact of PG with CD1b in cells (Fig. 1).

### A hierarchy of TCR affinity toward CD1b-phospholipid complexes

**Fig. 3.**
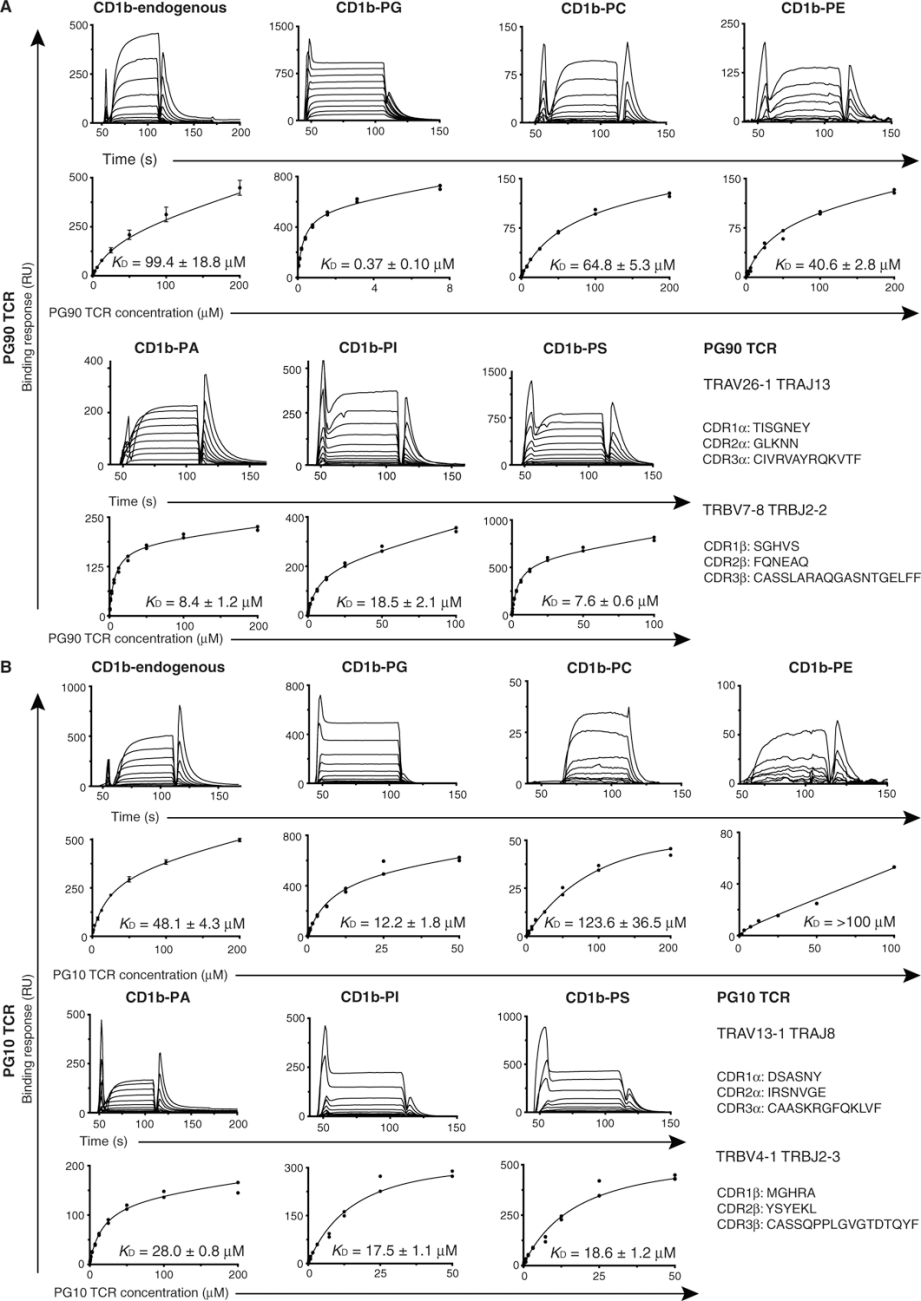
Steady-state affinity measurements of TCRs against CD1b-presenting phospholipids. SPR steady-state affinity measurements for the (**A**) PG90 and (**B**) PG10 TCRs, against CD1b presenting a range of phospholipid ligands, including CD1b in complex with endogenous lipids (CD1b-endo), CD1b-PG, CD1b-PC, CD1b-PE, CD1b-PI, CD1b-PA, and CD1b-PS. SPR experiments were conducted as duplicate in two independent experiments; CD1b-endo against PG10 and PG90 TCRs was carried out in three independent experiments. Steady-state *K*_D_ values (μM), as well as error bars, represent mean ± SEM. SPR sensograms (top) and equilibrium curves (bottom) were prepared in GraphPad Prism 7.0. For equilibrium curves for which *n* ≤ 2, data points from both independent experiments are presented with no error bars. The (A) PG90 and (B) PG10 TCR CDR amino acid loop sequences and gene usage are included. See table S3 for raw data.

We expressed, refolded, and purified the PG90 TCR (TRAV26–1 and TRBV7–8) and the PG10 TCR (TRAV13–1 and TRBV4–1) to homogeneity and measured, via surface plasmon resonance (SPR), their steady-state affinity (*K*_D_) toward CD1b proteins loaded with diverse endogenous lipids captured in cells (CD1b-endo) (Fig. 3). The affinity of the PG90 TCR and the PG10 TCR toward CD1b-endo expressed in HEK 293S cells is 99.4 and 48.1 μM, respectively (Fig. 3, A and B).

We measured the effects of PG, PS, PA, PE, PI, and PC on these CD1b-TCR interactions, where the phospholipids were specifically loaded onto CD1b and subsequently purified. The shift in the isoelectric focusing gel and anion exchange purification of the CD1b-phospholipid complex shows that the phospholipids are loaded into CD1b at high occupancy and purified to homogeneity (fig. S7). These lipids represent abundant (PI, PE, and PC), intermediate (PA), or rare (PS and PG) self-phospholipids in cells. When diversely loaded CD1b-endo complexes are treated with defined lipids, the TCR affinities are markedly changed, allowing ranking of lipids for mediating TCR binding. For the PG90 TCR, the affinity ranking is PG > PS ≈ PA > PI > PE > PC > endo (Fig. 3A). For the PG10 TCR, a similar hierarchy was observed: PG > PI ≈ PS > PA > endo > PC >> PE (Fig. 3B). Thus, the TCR is sufficient to alter the reactivity profiles toward CD1b-lipid complexes composed of phospholipids that all share a core PA structure. The PG10 TCR affinity for CD1b-PG is 12.2 μM, which was higher but comparable with that of PI (17.5 μM), PS (18.6 μM), and PA (28.0 μM) and notably greater than the other CD1b-phospholipid complexes (Fig. 3B). In contrast, the PG90 TCR affinity for CD1b-PG (0.37 μM) was ~300-fold higher than that of CD1b-endo, a value that is notably high for self-ligand recognition (Fig. 3A) (1).

### A central TCR footprint on CD1b

**Fig. 4.**
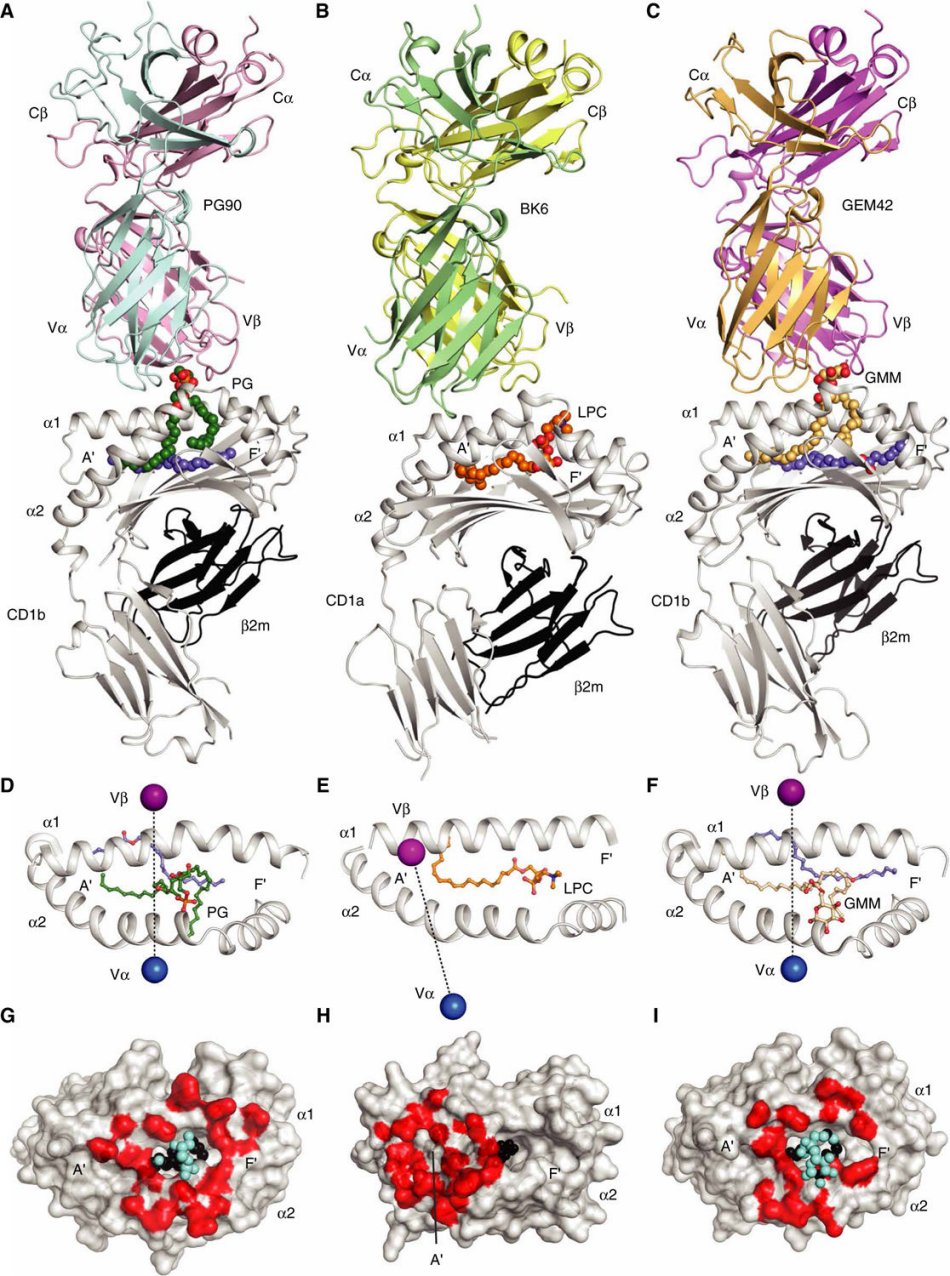
Comparison of ternary TCR-lipid-CD1 complex structures. Crystal structures of self-lipid presentation by CD1b (PG90 TCR-CD1b-PG) (**A**, **D**, and **G**) and CD1a [BK6 TCR–CD1a–lysophosphatidylcholine (LPC)] (**B**, **E**, and **H**) (29) and foreign antigen presentation by CD1b (GEM42 TCR-CD1b-GMM) (**C**, **F**, and **I**) (16). Blue and purple spheres, representing the center of mass of TCR α and β, respectively (D to F), footprints (red, bottom panels), and extent of antigen interaction (cyan), with antigen atoms not involved in the interaction in black (G to I). Ternary complexes (A to C) are shown in ribbon diagrams and bound lipid antigens as spheres. Blue, scaffold lipids; dark green, PG; orange, LPC; yellow, GMM.

We solved the structures of CD1b-PG and the PG90 TCR-CD1b-PG complex (fig. S8 and table S1), permitting comparisons with previously determined TCR-lipid-CD1 ternary complexes (Fig. 4, A to I, and table S2) (16, 29). The PG90 TCR docked approximately orthogonally over the long axis of the CD1b antigen-binding cleft, whereupon the TCR α and β chains docked over the α2 and α1 helices of CD1b, respectively (Fig. 4, A, D, and G). The PG90 TCR was positioned centrally atop CD1b, where it made extensive contacts with the phosphoglycerol head group, forming a total buried surface area (BSA) of approximately 1800 Å^2^ (Figs. 4, A, D, and G, and 5A). This mode of CD1b binding and antigen recognition contrasted with that of a CD1a-autoreactive TCR known as BK6. The ectopic, left-sided TCR footprint of BK6 did not contact lipid antigens positioned within or on the right side of the CD1a platform, explaining the observed low specificity for differing CD1a autoantigens (Fig. 4, B, E, and H) (29). The more right-shifted footprints of the GEM42 (Fig. 4, C, F, and I) and PG90 TCRs on CD1b (Fig. 4, A, D, and G) allow the TCR to fully surround the protruding ligand, forming many contacts that mediate high TCR specificity for GMM and PG, respectively (16).

### Mechanism of CD1b-phospholipid recognition

**Fig. 5.**
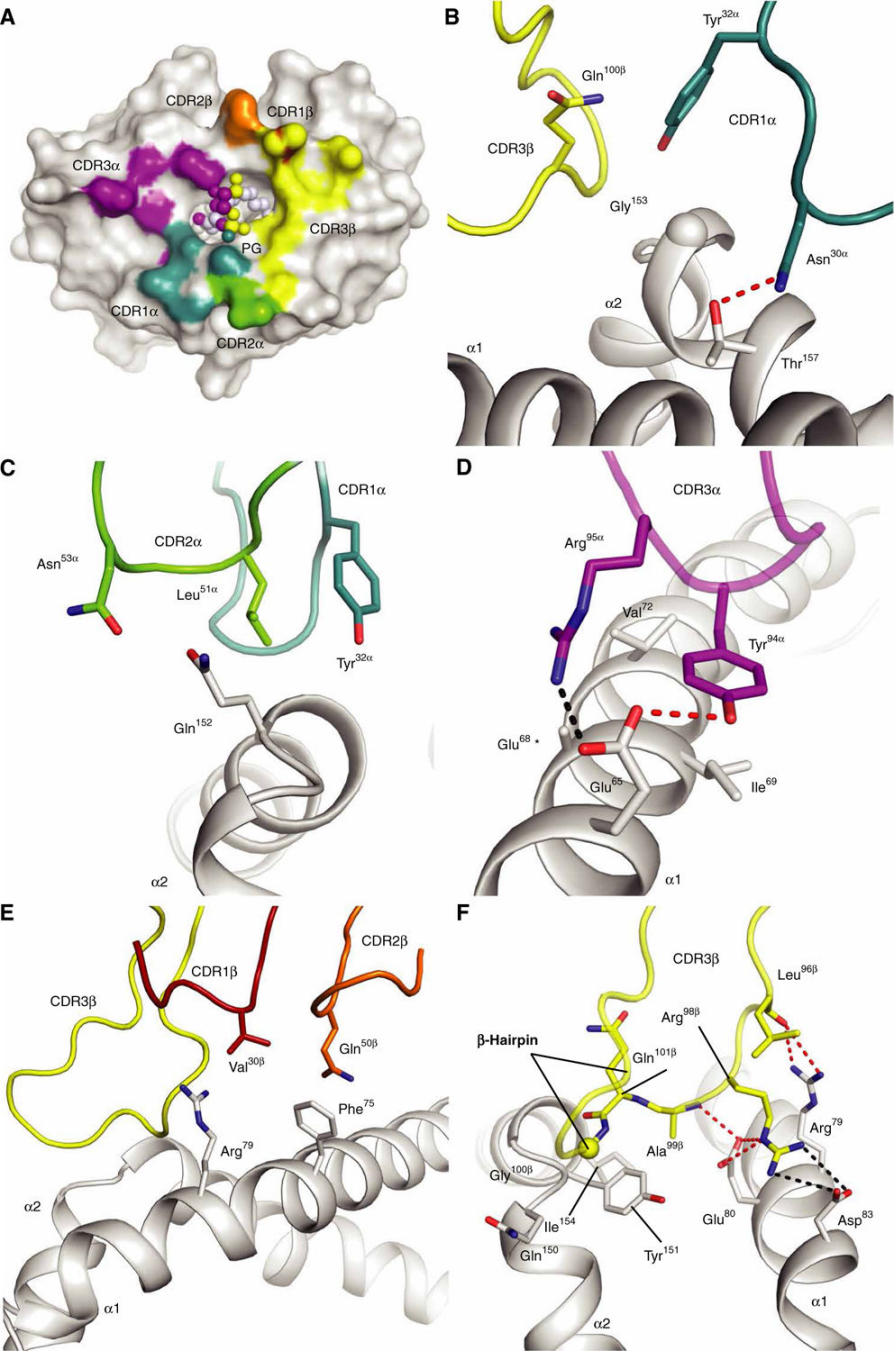
Docking footprint of PG90 TCR on CD1b-PG. (**A**) Footprint of PG90 CDR loops on CD1b. CD1b is represented as a white surface, with PG represented as spheres. Points of contact with TCR CDRs shown in the indicated color. Contacts between (**B**) CDR1α (teal), (**C**) CDR2α (green), (**D**) CDR3α (purple), (**E**) CDR1β (red) and CDR2β (orange), and (**F**) CDR3β (yellow) and CD1b are highlighted. Hydrogen bonds and salt bridges are represented as red and black dashes, respectively. Amino acid residues are represented as sticks, with the exception of glycine residues, which are represented as spheres. The spheres in (B) and (F) represent the Cα atoms of the glycine residues. No side chain is represented for residue Glu^68^ (*) because of the lack of electron density.

Apart from complementarity-determining region 1β (CDR1β), all CDR loops contributed notably toward contacts with CD1b-PG (Fig. 5A and table S2). Here, the CDR3α (270 Å^2^) and CDR3β (410 Å^2^) loops were the principal contributors, providing 26 and 40% of the BSA, respectively (Fig. 5A), followed by the CDR1α (120 Å^2^; 12% BSA), CDR2α (100 Å^2^; 10% BSA), and CDR2β (90 Å^2^; 9% BSA) loops. The CDR1β loop chiefly interacted with CD1b via Asn^30α^ hydrogen bonding to Thr^157^ of CD1b, whereas Tyr^32α^ abutted Gln^100β^ from the CDR3β loop and made van der Waals interactions with Gly^153^ CD1b (Fig. 5B). Immediately adjacent to Tyr^32α^ is Leu^51α^from, which, together with Asn^53α^, partially enveloped Gln^152^ from CD1b (Fig. 5C). The footprint the CDR3α loop made on the α1 helix of CD1b is expansive (Fig. 5A), which is principally related to the bulky side chains of Tyr^94α^ and Arg^95α^ converging onto a constellation of residues spanning positions 65 to 72 of CD1b (table S2), whereupon a mixture of polar and nonpolar interactions is formed with Glu^65^, Glu^68^, Ile^69^, and Val^72^ (Fig. 5D).

The germline-encoded interactions between the TRBV7–8 chain of the PG90 TCR were suboptimal in engaging CD1b, where the interactions were largely limited to the protruding Phe^75^ and Arg^79^ from CD1b, making peripheral van der Waals interactions with Gln^50β^ and Val^30β^, respectively (Fig. 5E). Instead, the 17–amino acid–long CDR3β loop bridged the portal to the antigen-binding cleft of CD1b and interacted with the PG ligand itself, as detailed below (Fig. 5A). Here, Arg^98β^ interdigitated between a network of charged residues on CD1b, packing against Arg^79^ and forming salt-bridging contacts with Glu^80^ and Asp^83^, which collectively formed a complementary suite of interactions (Fig. 5F). These contacts are extended by a β-hairpin loop structure (residues Ala^99β^-Gly^106β^), which enabled the CDR3β loop to extend away from the antigen-binding cleft and make main-chain backbone interactions against residues within the α2 helix of CD1b (table S2). Overall, comparison of the germline-encoded and the non-germline-encoded contributions of the PG90 TCR suggests that the CDR3 loops are the main driving factors enabling the autoreactivity toward CD1b.

### Key CD1b residues mediating TCR recognition

**Fig. 6.**
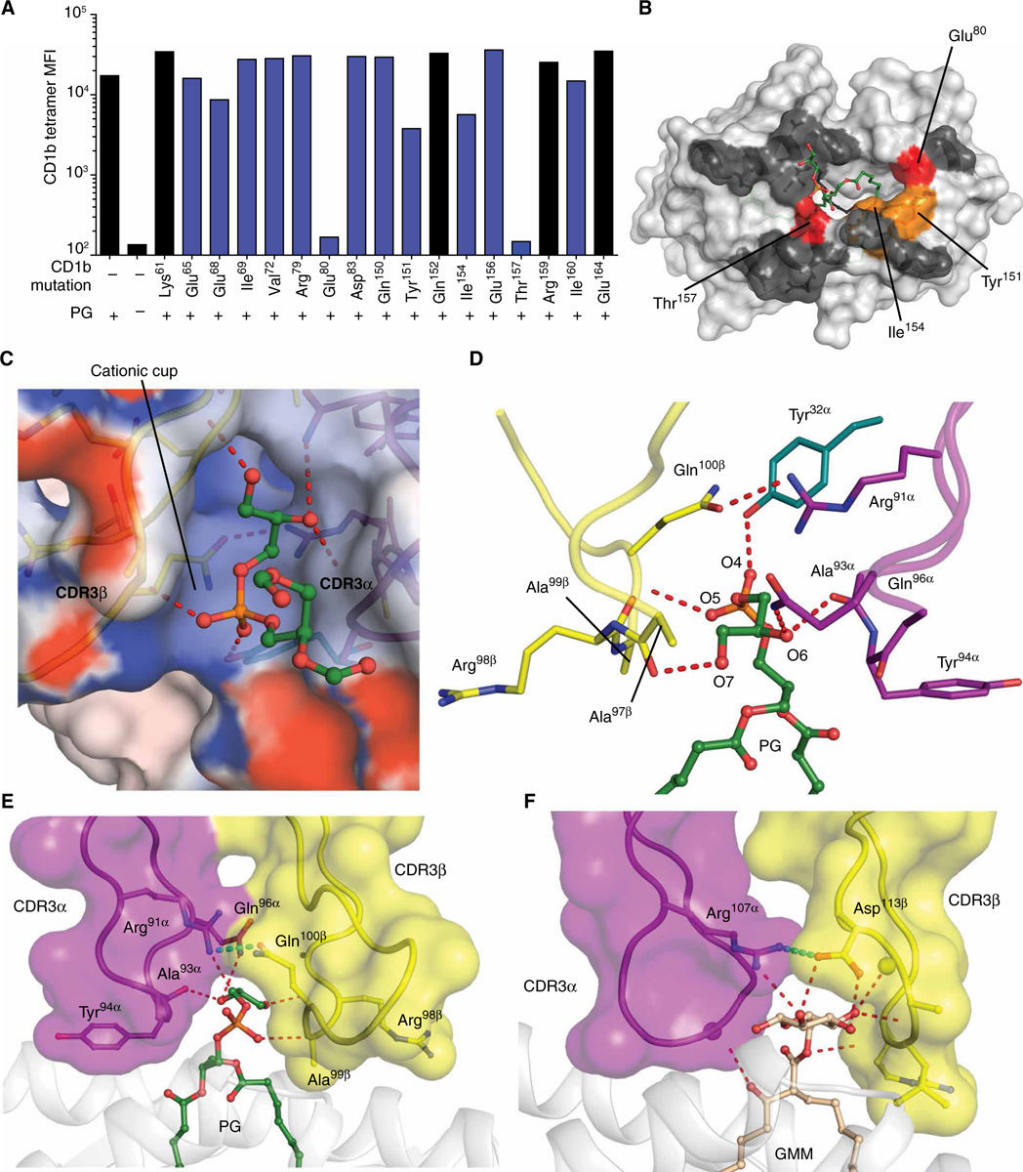
The functional and structural basis of phospholipid recognition by the PG90 TCR. (**A**) Staining of the PG90 cell line by CD1b tetramers with a single alanine mutant at the indicated site with or without bound PG. Residues interacting with the PG90 TCR are highlighted in blue. MFI, mean fluorescence intensity. (**B**) Color coding summary: white, not tested; dark gray, no effect (<25% reduction); orange, moderate effect (25 to 75% reduction); red, markedly reduced binding (>75% reduction). (**C**) Electrostatic potential of the cationic cup. The potential contours are shown on a scale from +10.0 (positive charge, blue) to −10.0 *k*_B_*T*e^−1^ (negative charge, red); white indicates a value close to 0*k*_B_*T*e^−1^ (neutral charge). (**D**) Side view of hydrogen bond network between PG (green) and PG90 TCR, with CDR1α, CDR3α, and CDR3β loops represented in teal, purple, and yellow, respectively. Contacts with the head group of PG (**E**) and GMM (**F**). The interactions are represented as red dashes, whereas the cyan dash represents the hydrogen bond (E), and salt bridge (F), that forms the arginine capstone.

Next, we evaluated the function of individual CD1b residues in TCR binding through alanine mutation of residues positioned on the outer surface of the α1 helix (positions 61, 65, 68, 69, 72, 79, 80, and 83) and the α2 helix (positions 150, 151, 152, 154, 156, 157, 159, 160, and 164), which included residues that did or did not contact the PG90 TCR (table S2). CD1b monomers were tetramerized, loaded with PG, and tested on the PG90 T cell line. In agreement with the SPR measurements (Fig. 3), tetramer staining was dependent on PG, which increased staining by ~100-fold, providing a large dynamic range to assess both strong and weak effects of individual mutations. Here, strong effects at Glu^80^ and Thr^157^ and weaker effects at Ile^154^ and Tyr^151^ were observed (Fig. 6A), which corresponded to interaction sites with the CDR3α and CDR3β loops, as well as the CDR1α loop (Figs. 5, B, D, and F, and 6B), indicating that these loops functionally dominate the interaction.

### TCR specificity for the phosphoglycerol head group

The PG90 TCR binds to and recognizes the PG antigen with high affinity and specificity (Figs. 1 to 3), and the ternary complex provides immediate insight into these characteristics. The PG90 TCR completely sequesters the phosphoglycerol head group such that it is no longer solvent-exposed after TCR engagement. Here, the electronegative phosphate ester was inserted into a “cationic cup” with a positively charged environment formed by the CDR1α, CDR3 α, and CDR3β loops (Fig. 6C). Although Arg^91α^ is the only positively charged residue within this cationic cup, the orientation of the amide backbone atoms alongside the exclusion of any acidic residues within this cup imparts the clear electropositive potential (Fig. 6C). The phosphoester moiety hydrogen bonds to Tyr^32α^ while also forming van der Waals interactions and additional hydrogen bonds with the main chains of the CDR3α and CDR3β loops, as well as packing against Gln^100β^ (Fig. 6D). The distal glycerol moiety of PG snakes deeply into the TCR pocket, between TCR α and β, forming hydrogen bonds with Gln^96α^ and the main chain of the CDR3β loop and van der Waals contacts with Arg^91α^ (Fig. 6E). This snug fit of the distal glycerol components of the PG ligand between the TCR chains correlated well with the observed specificity of the PG90 TCR for PG in preference to other phospholipids that contained the same PA core structure but lacked the glycerol moiety (Figs. 1 and 3).

Structural understanding of TCR binding to CD1b is currently limited to the PG90 and GEM42 TCRs (Figs. 4 and 6, E and F) (16). Despite the differing recognition requirements of glycolipid and phospholipid antigens, respectively, we could identify two mechanistic commonalities. For the PG90 TCR, Arg^91α^ from the CDR3α loop interacts with both the TCR β chain at Gln^100β^ and the phospholipid head group of the antigen (Fig. 6E). This dual role in TCR pairing and antigen recognition is an analogous role to the “Arg^107α^ capstone” in the GEM42 TCR (Fig. 6F). In addition, both PG90 and GEM42 TCRs fully surround the head group using a “tweezers grip” to extensively interact with exposed polar moieties of antigen head groups.

### TCR induced fit

**Fig. 7.**
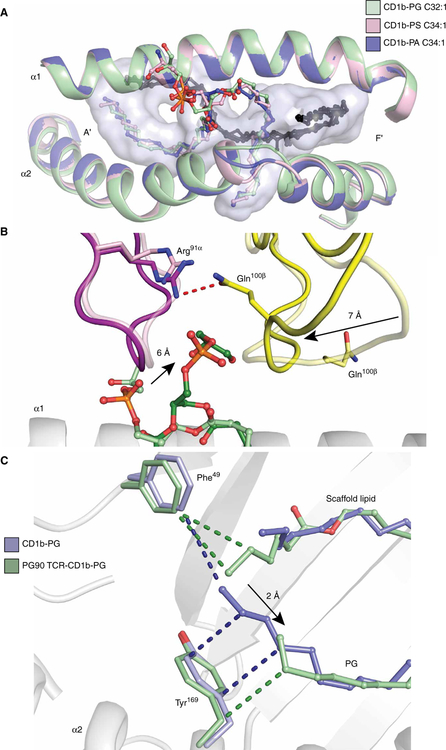
CD1b presentation of phospholipids and movement of PG upon PG90 TCR docking. (**A**) Top view shows superposition of CD1b-PG (green), CD1b-PA (dark blue), and CD1b-PS (pink), with scaffold lipids colored black. Oxygen, nitrogen, and phosphate groups are colored red, blue, and orange, respectively. The CD1b antigen-binding cleft is represented as a light blue transparent surface. (**B**) Overlay of the separately solved structures of CD1b-PG (light green) and the PG90 TCR (light purple and yellow) with the ternary CD1b-PG-PG90 TCR (dark green, dark purple, and dark yellow) structure. (**C**) CD1b is represented as a cartoon in white, and PG and scaffold lipids are represented as sticks in blue (CD1b-PG) and green (PG90 TCR-CD1b-PG). Hydrophobic interactions between Tyr^169^ and Phe^49^, and PG and scaffold lipids are represented as blue (CD1-PG) and green (PG90 TCR-CD1b-PG) dashes. Direction of movement is indicated by a black arrow.

To determine whether the differing TCR reactivity toward distinct phospholipid ligands could derive from conformational change of CD1b induced by the lipid, we also determined the structures of the PG90 TCR and the CD1b-PS and CD1b- PA complexes in their binary states (table S1 and figs. S8 and S9) and then compared them with the PG90 TCR-CD1b-PG ternary complex (Fig. 7). The refined 2*F*_o_ − *F*_c_ electron density maps showed clear positioning of the acyl tails but not of the PA and PS head groups (Fig. 7A and fig. S9). Because these binary complexes were solved at high resolution, the weak head group densities likely resulted from their lack of interaction with the CD1b molecule itself. The crystal structures of CD1b-PG, CD1b-PA, and CD1b-PS were very similar (Fig. 7A and fig. S9), revealing that the differing reactivities toward the phospholipids were not due to altered presentation of these ligands by CD1b or marked changes in CD1b itself.

However, the placement of the PG head group was markedly altered upon PG90 TCR docking, where the PG head group is shifted by 6.0 Å and locked between the CDR3 loops (Fig. 7B). The movement of the PG head group lifts the phospholipid slightly out of the antigen-binding cleft, resulting in a displacement of the sn2 fatty acid by about 2.0 Å compared with the CD1b-PG binary crystal structure (Fig. 7C). These conformational changes are also accompanied by movement in the PG90 TCR itself, whereby the CDR3β loop was repositioned by about 7.0 Å to make favorable contacts with the PG head group (Fig. 7B). Once PG is locked between the CDR3 loops, the phosphate head group is stabilized by an additional hydrogen bond by Tyr^32α^ on the CDR1α loop (Fig. 6D). A similar mechanism is observed in the case of the GEM42 TCR-CD1b-GMM crystal structure (fig.S10) (16). Accordingly, the antigen-binding pocket of the PG90 TCR is not preformed but is molded upon CD1b-phospholipid engagement.

Specificity for PG is driven through hydrogen bond formation against the glycerol moiety of the antigen head group by Gln^96α^ and the main chain of Ala^93α^ and Ala^97β^ (Fig. 6D and table S2). This docking mechanism can explain the observed hierarchy of phospholipid recognition (Fig. 3) based on the extent to which the head groups in each phospholipid class diverge from the size and electrochemical properties of glycerol. PG, PA, and PS are relatively conserved in the size of their head groups, so steric clashes with the TCR are not envisioned. The modestly lower TCR affinity for CD1b-PA is plausibly explained by its smaller head group and subsequent loss of interaction with Ala^97β^ and Ala^93α^ (Fig. 6D). Although the serine moiety in PS is similar in size to glycerol, the NH_3_ group might provide electrostatic repulsion within the PG90 TCR pocket (Fig. 6C). Other phospholipids contain head groups that are predicted to clash with the snug TCR pocket. Specifically, the choline and inositol head groups present in PC and PI (Fig. 1A) exceed the volume present distal to the cationic cup, located between the TCR chains. Further, PE and PC carry positively charged head groups that have electrochemical incompatibility with the cationic cup. Accordingly, it is the architecture of the cationic cup (Fig. 6, C and D) that simultaneously explains the specificity for PG recognition by PG90 and aids in explaining its markedly reduced affinity toward a number of other common cellular phospholipids.

## DISCUSSION

As contrasted with an understanding of the structural and selection mechanisms for limiting autoimmunity in the MHC system (30–33), evidence regarding control of T cell autoreactivity toward lipid antigens is just emerging (34–38). CD1-restricted T cell autoreactivity can manifest in a range of disease settings, including CD1b-mediated psoriasiform inflammation, CD1a-mediated contact hypersensitivities, allergic responses to bee venom and house dust mites, and psoriasis (21, 39–42). Mechanisms for regulating T cell autoreactivity to CD1 proteins and self-lipids remain to be established. Here, we describe a mechanism of antigen recognition in which TCRs recognize a rare self-antigen in preference to the much more common membrane phospholipids present in cells. A notable difference between the patterns of MHC-I and CD1b expression on peripheral cells in vivo is that the former is ubiquitously expressed on nearly all nucleated cells, whereas CD1b is inducibly expressed mainly on macrophages and myeloid DCs (43). Our data suggest that PG is a rare self-antigen that is normally expressed near the threshold of detection by autoreactive T cells. One possibility supported by this work is that the amount of CD1b and phospholipid antigen available for scanning by T cells is restricted in time and location.

The mode of CD1b-mediated autoreactivity observed here is in contrast to the patterns of CD1a-mediated autoreactivity described previously (29). Here, an autoreactive TCR contacted CD1a only and not the lipid autoantigen itself. Accordingly, the role of the self-lipid was to act through the absence of interference with CD1a-TCR contact (12, 29, 36). In contrast, this example of a human TCR bound to CD1b and a self-lipid establishes a “stereochemical complementarity” mechanism. Namely, a centrally located TCR footprint fully surrounds a protruding lipid head group, forming many highly specific and direct interactions with the phospholipid.

CD1a, CD1c, and CD1d were known to bind phospholipids (36, 44), and our data now show that CD1b binds to most major classes of endogenous self-phospholipids, where the phosphate ester is both the defining chemical element of these compounds and potentially an electrochemically dominant element for binding proteins. The TCR induces a dramatic conformational change, whereby the anionic phosphate ester is inserted into a cationic cup within the TCR. This induced fit forces the glycerol group that is distal to the phosphate into extensive contacts with the TCR within a constrained space. The most abundant self-membrane lipids in mammalian cells have the same core PA structure needed for binding to CD1b but are distinguished by their differing head groups, which can show steric or electrostatic incompatibilities with TCR regions distal to the cationic cup. Therefore, this stereochemical complementary model provides an explanation for how T cells could distinguish among the major classes of cellular phospholipids in cells.

In considering their biological roles, it is notable that cellular phospholipids are targets of clonotypic receptors on both B cells and T cells. Immunologists have studied anti-phospholipid antibodies for decades because they cause hypercoagulable states in systemic autoimmune diseases (25). CD1d presentation of self-phospholipids to NKT cells has been observed in several studies and has been linked to anti-phospholipid antibody formation in mice (45, 46). For CD1b, evidence of a biological role for phospholipids in T cell response emerged only recently from studies that identified phospholipids as targets of inflammation ex vivo or in vivo (19, 21). Because CD1b and cellular phospholipids are expressed by macrophages and myeloid DCs in vivo, basic questions arise regarding control of potentially deleterious T cell reactivity that could cause autoimmunity. Our work provides mechanistic glimpses into autoantigen recognition by human TCRs, establishing stereochemical complementarity as the mechanism for discriminating among classes of self-phospholipids.

## MATERIALS AND METHODS

### Study design

The overall objective of the study was to determine the basis of TCR recognition of phospholipids presented by CD1b. To enable this goal, we undertook experiments centered around cellular immunology, MS, protein chemistry, SPR binding studies, and protein x-ray crystallography. The number of independent experiments is outlined in the figure legends, where appropriate.

### Lipid antigens

Synthetic C32:1 PA (#840857), PE (#850757), PS (#840034), PI (#850142), and PC (#850457) were purchased from Avanti Polar Lipids. PG extract from *Escherichia coli* (#841188) was purchased from Avanti Polar Lipids. *E. coli*–derived C18:1 lyso-sulfatide (#1904) was purchased from Matreya.

### Lipid extraction

Each protein sample (CD1b-endo, CD1b-PG, and CD1b-PG-PG90) was transferred to a 15-ml glass tube and extracted with chloroform, methanol, and water according to the method of Bligh and Dyer (47). The resulting two-layer mixture was centrifuged at 2000 rpm for 10 min. The lower organic lipid–containing layer was collected to another clean glass tube, and the organic solvents were dried under nitrogen gas. The lipid residue was redissolved in chloroform/ methanol (1:2) to a normalized concentration of 5 μM based on the quantity of the input protein. For cells, C1R, K562, and G4 monocytes (~50 million cells each) were harvested from cell cultures. The cells were washed with phosphate-buffered saline twice and extracted with 1:2 (v/v) chloroform/methanol for 2 hours, followed by 2:1 (v/v) chloroform/methanol for another 2 hours. Lipid extractions were combined and dried under nitrogen gas. Lipids (~0.6- to 2-mg yield) were redissolved in chloroform/methanol (1 mg/ml) and stored at −20°C.

### Lipid identification by MS

To inspect all lipids without column separation, lipid extracts (~10 μl) were loaded onto a nanospray tip for ESI-MS and multistage collision-induced dissociation tandem MS using a two-dimensional ion trap mass spectrometer (LXQ, Thermo Fisher Scientific). Collision energy was 20 to 30% of the maximum, and product ions were trapped with a *q* value of 0.25. To gather the retention time and accurate mass information, the extracted lipids were analyzed by HPLC-ESI-QToF (Agilent Technologies 6520 Accurate-Mass Quadrupole Time-of-Flight coupled with an Agilent 1200 Series HPLC) using a Varian MonoChrom Diol column (3 μm × 150 mm × 2 mm) and a Varian MonoChrom Diol guard column (3 μm × 4.6 mm) according to the published methods (48). Cellular lipids were dried and redissolved in the starting mobile phase solvents of 70:30 hexane/isopropanol at 1 mg/ml to allow injection of 20 μl. For lipids eluted from CD1b or other defined proteins, input lipid was normalized to the mass of protein treated (25 μg). PC, PE, and PG concentrations in each sample were calculated by comparing the peak areas of the extracted ion chromatograms with a separately generated standard curve. Chain length analogs were assumed to have equivalent ionization response factors. To estimate the percentage of each lipid family among total extracted cellular lipids, the absolute concentrations calculated for every detected chain length analog peak within each family were summed and then divided by the total amount of lipids injected. Lipid standards (PC, catalog no. 770365; PG, catalog no. 840503; PE, catalog no. 850757) were purchased from Avanti Polar Lipids.

### TCR protein expression and purification

Recombinant PG90, PG10, and GEM42 TCRs were cloned with a BirA tag (amino acid sequence, GLNDIFEAQKIEWHE) at the C terminus of the α chain, and were refolded essentially as per methods previously described (49). The PG90-BirA and GEM42-BirA proteins were biotinylated using recombinant BirA enzyme and excess D-biotin, as described (50). The level of biotinylation was validated by incubating purified biotinylated TCR with avidin and analyzed by native polyacrylamide gel electrophoresis.

### TCR tetramers

For tetramerization of biotinylated truncated TCR constructs, six aliquots of streptavidin-phycoerythrin conjugate of 0.35 μg each (S866, Invitrogen) were added, with 10-min intervals, to 2 μg of biotinylated PG90 or GEM42 TCR for a final concentration of 80 ng/μl. Staining of APCs was performed at room temperature using 40 ng per well in a volume of 50 μl. Flow cytometric analysis was performed on a BD Fortessa using a 561-nm laser.

### CD1b protein expression and purification

CD1b was produced in either HEK 293S GnTI^−^ cells (American Type Culture Collection) or insect *Trichoplusia ni* High Five cell lines, as previously described (16). Mutants of CD1b were expressed and purified in HEK 293S GnTI^−^ cells, as described above. CD1b mutants were biotinylated as per the PG90-BirA TCR.

### Crystallization and structure determination

Crystals of CD1b-phospholipid complexes were grown via the hanging drop vapor diffusion method, using a protein/reservoir drop ratio of 1:1, at a protein concentration of 5 mg/ml in 10 mM tris-HCl (pH 8.0) and 150 mM NaCl, with a reservoir solution of 20 to 30% (v/v) polyethylene glycol (PEG) 3350, 0.1 M sodium iodide, and 2% (v/v) propylene glycol. Crystals of the PG90 TCR were grown at a protein concentration of 8 mg/ml in 22% (v/v) PEG 3350 and 0.2 M magnesium formate. Crystals of the CD1b-PG-PG90 TCR ternary complex were grown at a protein concentration of 2 mg/ml, with a reservoir solution of 8 to 14% (v/v) PEG 8000, 0.1 M MES (pH 6.0), and 0.2 M zinc acetate.

Crystals were soaked in a cryoprotectant of mother liquor containing 30% (v/v) PEG 3350 for the CD1b-phospholipid and the PG90 TCR crystals and 20% ethylene glycol for the PG90-CD1b-PG ternary complex crystals before being flash-frozen in liquid nitrogen, and data were collected at the Australian Synchrotron. Data were processed using the iMosflm software and scaled with the Aimless software as part of the CCP4 suite (51). The structures were solved by molecular replacement using Phaser as part of the Phenix suite (52). Ligand electron density in the CD1b-phospholipid complexes for acyl tails was well defined; however, the phosphoester groups had weak electron density around the ligand head groups. Ligand electron density in the PG90 TCR-CD1b-PG complex was well defined. Amino acid side chains that are not visible in the electron density for all structures have been removed. Manual adjustment of the models was conducted using the Coot software (53), followed by maximum-likelihood refinement with phenix-refine. All molecular graphics representations were generated in PyMOL (54). BSAs were calculated using CASTp (55), and contacts were generated using the CONTACT program in the CCP4i program suite.

### Surface plasmon resonance

SPR analysis was conducted on the BIAcore 3000 instrument at 25°C in 10 mM tris-HCl (pH 8.0), 150 mM NaCl, and 1% (w/v) bovine serum albumin, as described previously (49). Experiments were conducted in duplicate or triplicate (*n* = 2 or 3), with wild-type CD1b-endo and CD1b-PG used as controls and HLA-B8-FLR used as the blank. Data analysis and visualization were conducted with GraphPad Prism 6.0 using the 1:1 Langmuir binding model.

## Supplementary Material

Supplement

Table S3
