# Supplementary material for: A molecular basis of human T cell receptor autoreactivity toward self-phospholipids
Source: Sci Immunol. Author manuscript; Available in PMC 2019 Jul 23. (PMC6649662; doi:10.1126/sciimmunol.aao1384)
Supplement: Supplement [file NIHMS1034873-supplement-Supplement.pdf]

immunology.sciencemag.org/cgi/content/full/2/16/eaao1384/DC1

Science Immunology

Supplementary Materials for

A molecular basis of human T cell receptor autoreactivity toward self-phospholipids

Adam Shahine, Ildiko Van Rhijn, Tan-Yun Cheng, Sarah Iwany, Stephanie Gras,*
D. Branch Moody,* Jamie Rossjohn*

*Corresponding author. Email: stephanie.gras@monash.edu (S.G.); bmoody@partners.org (D.B.M.); jamie.rossjohn@monash.edu (J.R.)

Published 20 October 2017, *Sci. Immunol.* **2**, eaao1384 (2017)
DOI: 10.1126/sciimmunol.aao1384

The PDF file includes:

Fig. S1. TCR tetramer validation.
Fig. S2. Representative gating strategy for Fig. 1B.
Fig. S3. Major lipid classes produced by C1R and K562 cells.
Fig. S4. TLC-MS analysis of C1R lipids nearly comigrating with a PG standard.
Fig. S5. Quantification of PG in cells or eluted from CD1b.
Fig. S6. Nanoelectrospray analysis of protein eluates.
Fig. S7. Loading of phospholipids in CD1b molecule.
Fig. S8. Electron density maps for the phospholipid antigens presented by CD1b.
Fig. S9. Phospholipid anchoring residues within CD1b pockets.
Fig. S10. Sequence alignments of TCR Vα and Vβ regions.
Table S1. Data collection and refinement statistics.
Table S2. Contacts between PG90 TCR and CD1b-PG.

Other Supplementary Material for this manuscript includes the following:
(available at immunology.sciencemag.org/cgi/content/full/2/16/eaao1384/DC1)

Table S3 (Microsoft Excel format). Raw data for Fig. 3A.

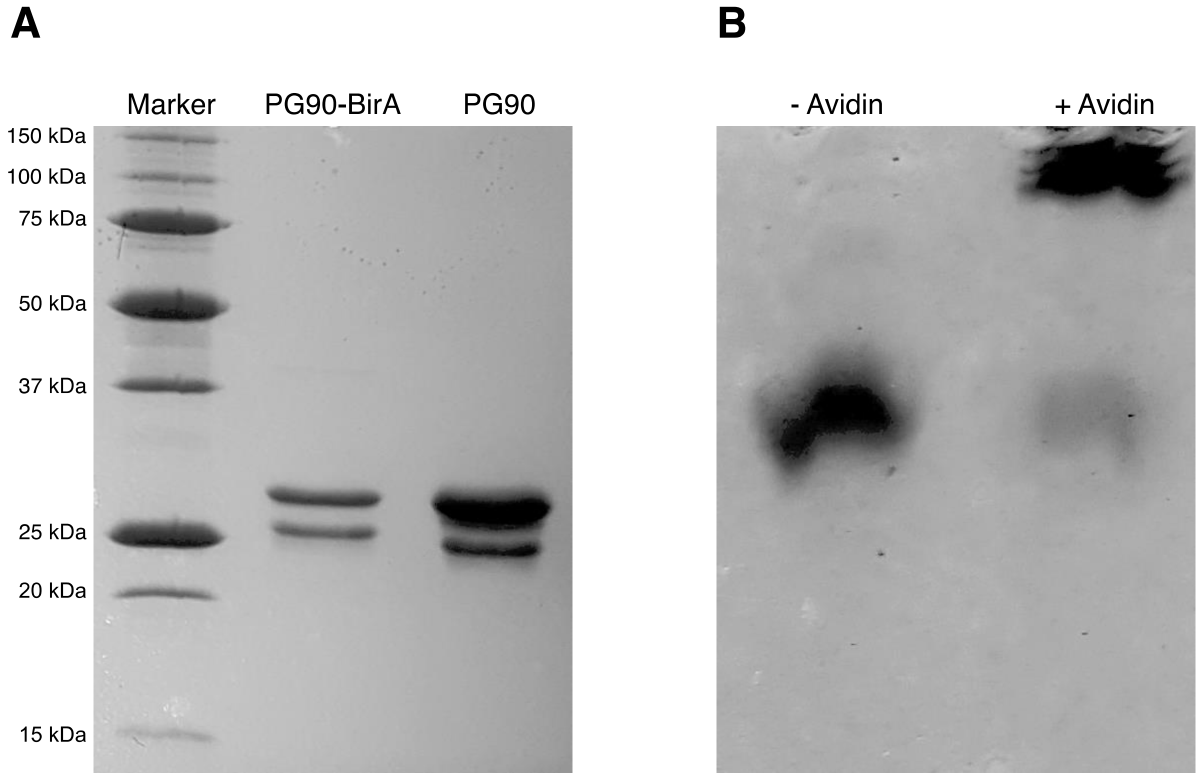

Fig. S1. TCR tetramer validation. (**A**) Polyacrylamide gel electrophoresis analysis (PAGE) of purified recombinant PG90 TCR and PG90 TCR biotinylated with a BirA sequence in a reduced state, visualising the α-chain (lower band) and β-chain (upper band). Protein markers and marker sizes are indicated in kilodaltons (kDa) (**B**) Native-PAGE of the biotinylated PG90-BirA monomer with and without with avidin.

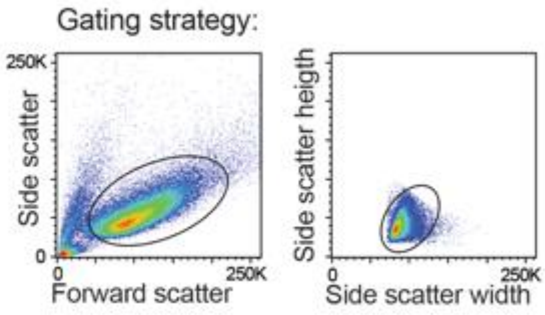

Fig. S2. Representative gating strategy for Fig. 1B. All events shown in Figure 1B were gated based on Forward and Side scatter to enrich for intact cells (left panel) and Side scatter width and height (right panel) to enrich for single cells.

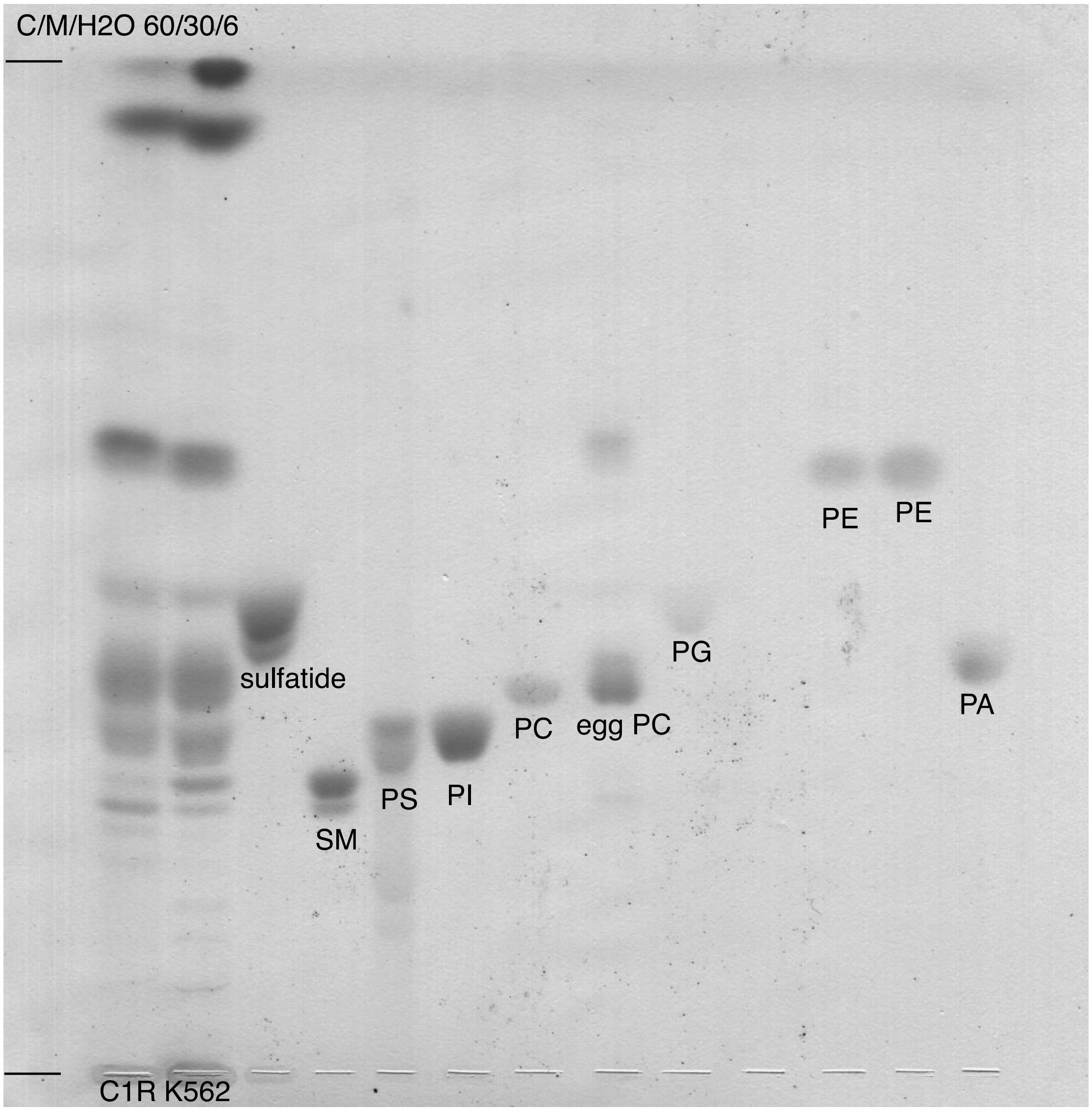

Fig. S3. Major lipid classes produced by C1R and K562 cells. Total lipid extracts (200 µg) from C1R and K562 cells and commercial lipid standards (10-20 µg) were loaded onto silica TLC plates and resolved in a solvent mixture of chloroform: methanol: water (60: 30: 6, V:V:V) and subjected to charring with 8% (V:V) phosphoric acid and 3% (W:V) cupric acetate. Sulfatide was from Matreya (10 µg, #1049) and other standards were purchased from Avanti polar lipids, which were sphingomyelin (SM, 10 µg, #860063), PS, phosphatidylserine (PS, 10 µg, #840032), phosphatidylinositol (PI, 10 µg, #840042), phosphatidylcholine (PC 10 µg, #850475), egg PC (10 µg, #840051), phosphatidylglycerol (PG, 10 µg, #840503), phosphatidylethanolamine, (PE, 10 µg and 20 µg, #850757), phosphatidic acid (PA, 10 µg, #840857). The lipid nearly co-migrating with PG and sulfatide was further analysed by TLC-MS (**Fig. S4**).

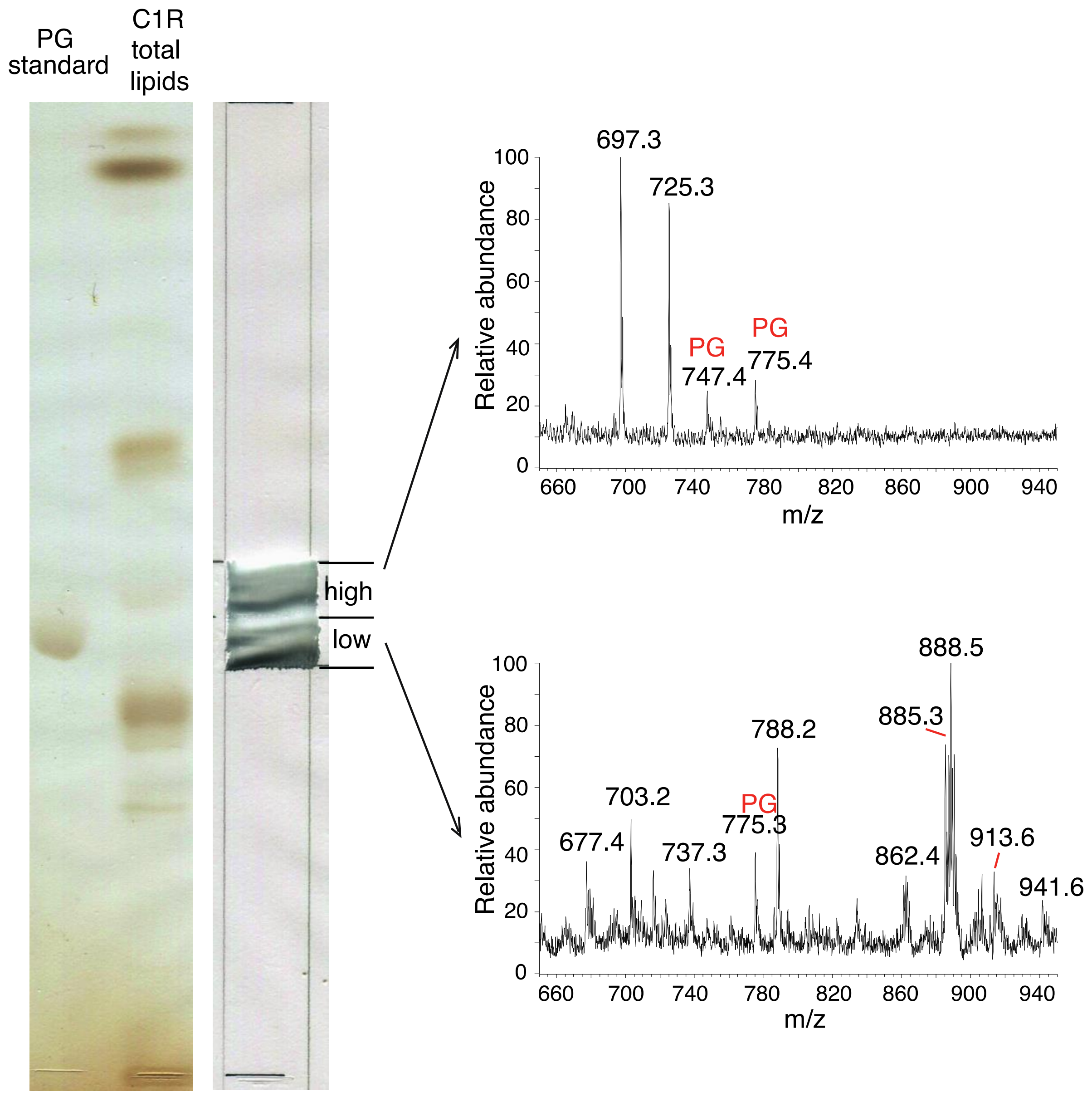

Fig. S4. TLC-MS analysis of C1R lipids nearly comigrating with a PG standard. Comparing charred lipids (left) to uncharred lipids (right) from C1R cells, silica was scraped at a retention time that matched the PG standard (low) or the most clearly visible band comprised of unknown lipids (high). Both were extracted in and subject to positive mode nanoelectrospray MS. Ions matching the known mass of PG are shown with red labels (PG), and other indicated *m/z* values do not match common PG species.

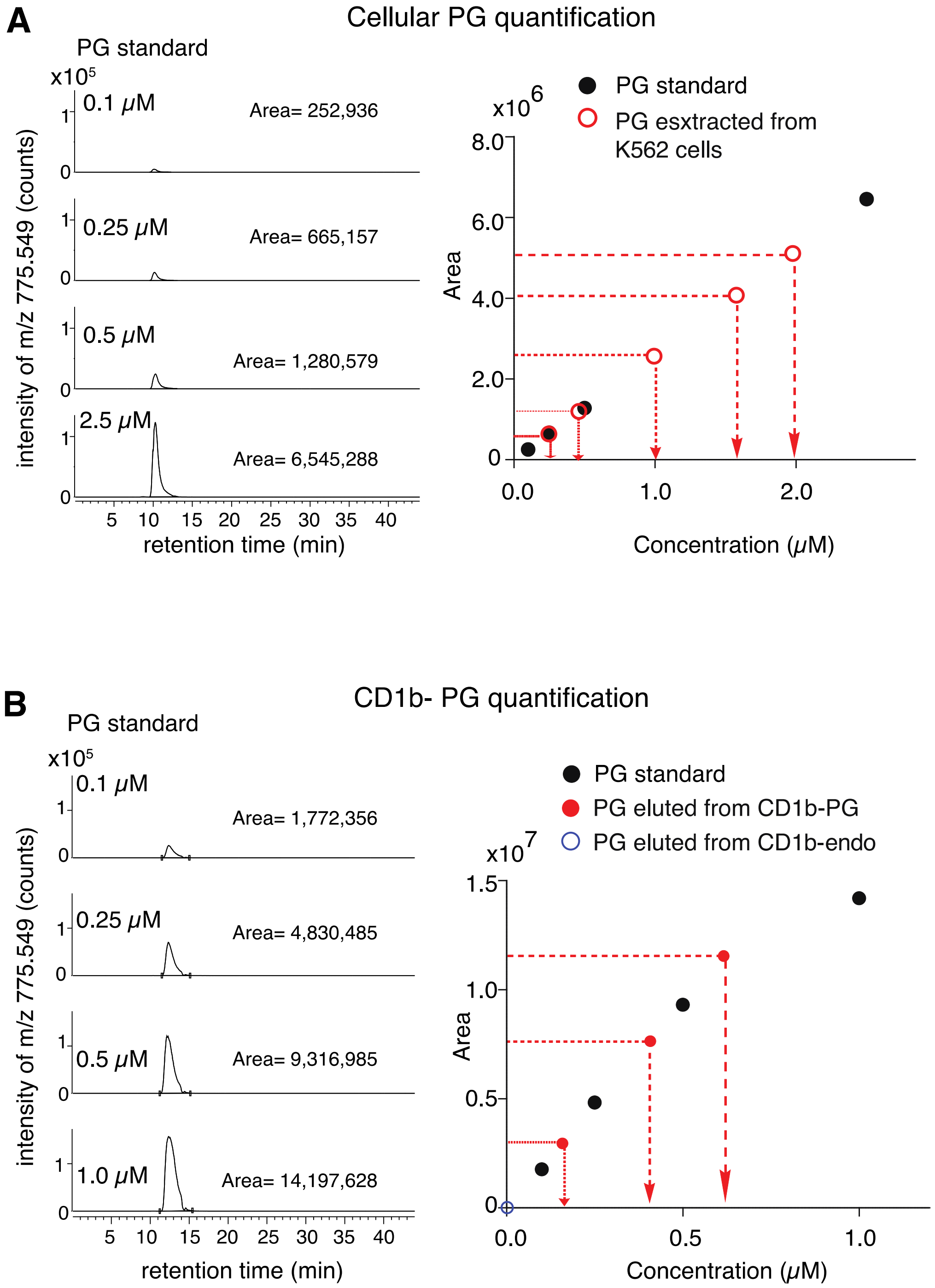

Fig. S5. Quantification of PG in cells or eluted from CD1b. A series of standard PG concentrations were prepared and analyzed by HPLC-MS (left panels). The peak areas of extracted ion chromatograms from known concentrations of external standards measured in count-seconds were used to generate a standard curve (black circles), which was used to

determine the concentrations of PG within K562 cells (**A**) or PG eluted from CD1b protein (**B**). The same approach was used to estimate absolute quantitation of PC and PE.

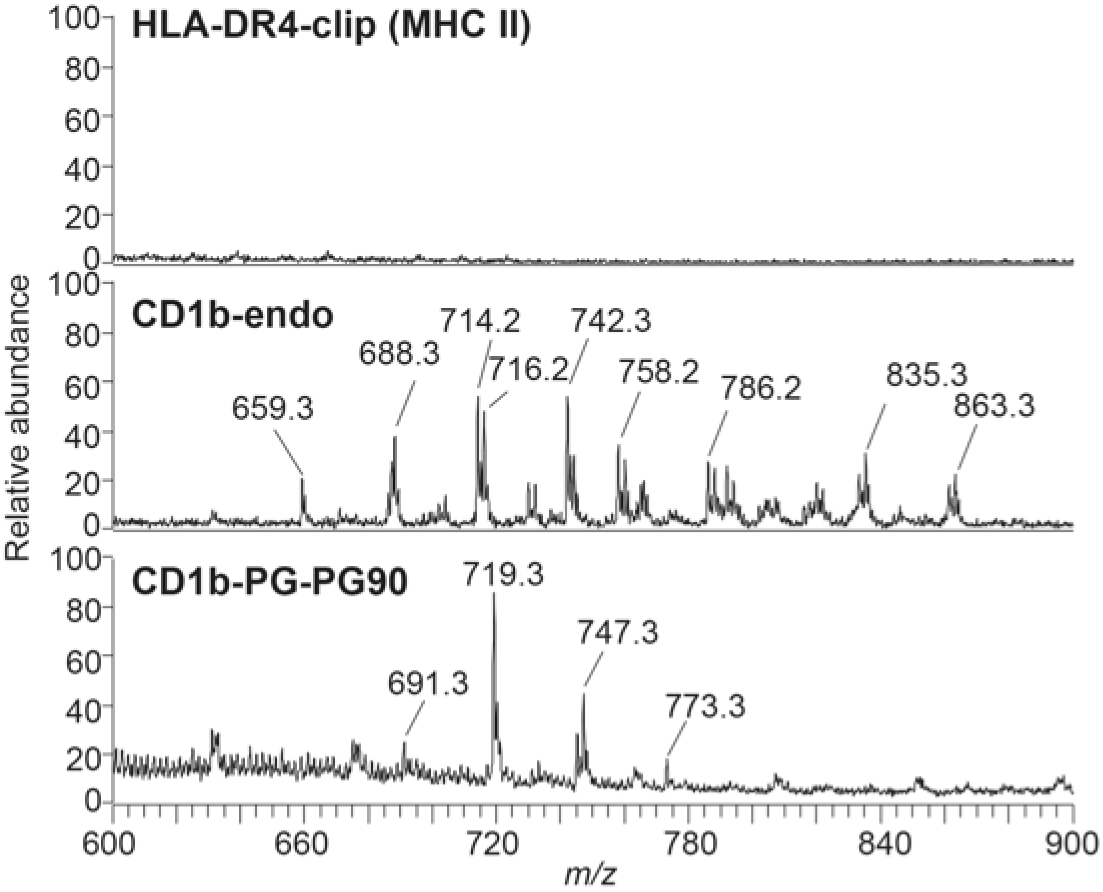

Fig. S6. Nanoelectrospray analysis of protein eluates. The lipid containing eluates from PG90 TCR-CD1b-PG crystals, CD1b carrying endogenous lipids and HLA-DR4-CLIP, which was used as a negative control protein for a protein lacking a lipid binding cleft, were normalized to the input proteins mass (5 µM) and analysed by positive mode nanoelectrospray with an ion trap MS detector.

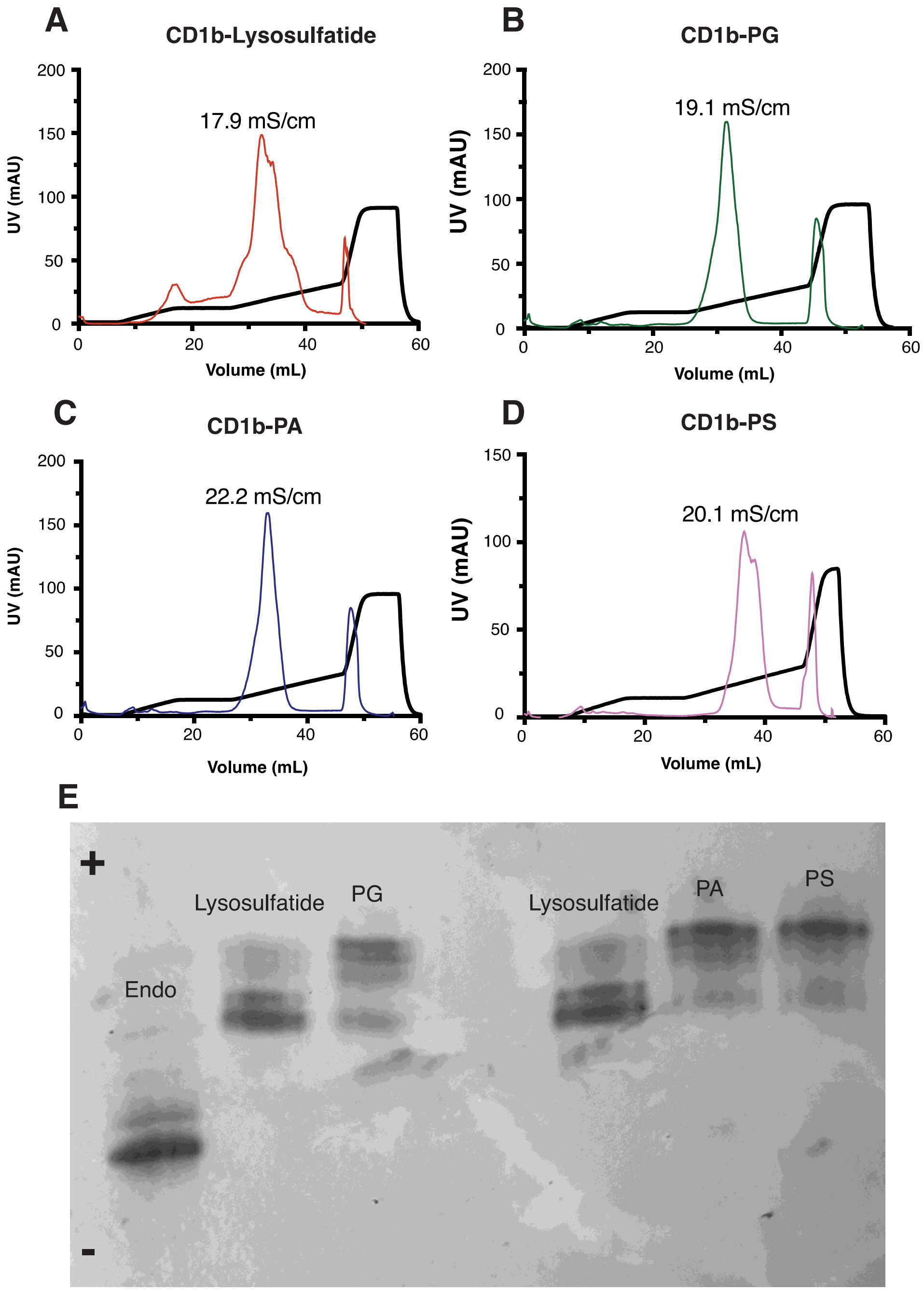

Fig. S7. Loading of phospholipids in CD1b molecule. CD1b was initially loaded with the charged lipid, lysosulfatide (A), and purified via anion exchange chromatography. The elution corresponding to CD1b loaded with lysosulfatide was subsequently loaded with PG (B), PA (C), and PS (D), before being purified again via anion exchange chromatography. Coloured plot represents the UV trace for each purification stage, with the black line representing the salt gradient, ranging from 0 M – 1 M NaCl. Conductivity reading (mS/cm) is indicated for each

peak corresponding to CD1b loaded with respective lipid. (E) Success of lipid loading into CD1b was validated via an isoelectric focusing (IEF) gel. CD1b was loaded onto the IEF gel and separated based on the pI of the protein; influenced by the charge of the bound lipid. Lanes corresponding to CD1b loaded with a specific lipid are indicated in the gel. Migration towards the positive cathode (+) indicates successful loading of the indicated lipid, in comparison to CD1b loaded with endogenous lipid mixture (Endo).

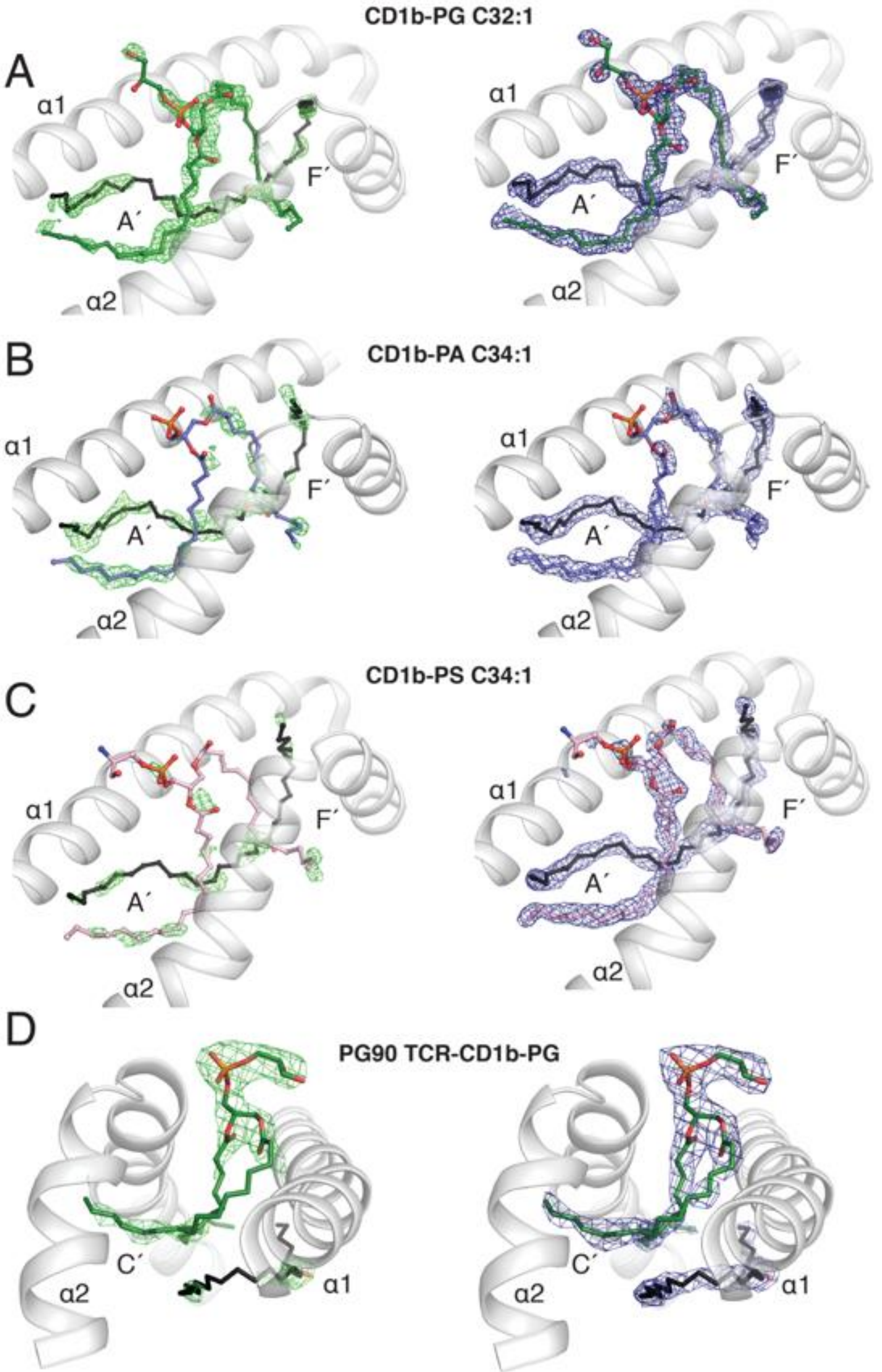
CD1b-PG C32:1
A
α1
F′
A′
α2
CD1b-PA C34:1
B
α1
F′
A′
α2
CD1b-PS C34:1
C
α1
F′
A′
α2
D
PG90 TCR-CD1b-PG
C′
α2
α1

Fig. S8. Electron density maps for the phospholipid antigens presented by CD1b. Lipids present in the antigen presenting grooves of the crystal structures of (**A**) CD1b-PG, (**B**) CD1b-PA, (**C**) CD1b-PS, and (**D**) PG90 TCR-CD1b-PG. Structures are visualised looking top down above the binding cleft of CD1b (**A-C**), and side view facing the A´-pocket (**D**). Lipids are represented as sticks and presented phospholipids are coloured (**A**) light green, (**B**) light orange, (**C**) light pink, and (**D**) dark green, with scaffold lipids in blue. Oxygen, nitrogen and phosphate are represented in red, blue and orange, respectively. Electron density maps are focused around both the phospholipids and scaffold lipids. Omit density maps (mFo-DFc) are contoured to 3.0 σ, with the exception of D, which is contoured to 2.0 σ, and coloured in green (left). Refined density maps (2mFo-DFc) are contoured to 1.0 σ are coloured in blue (right).

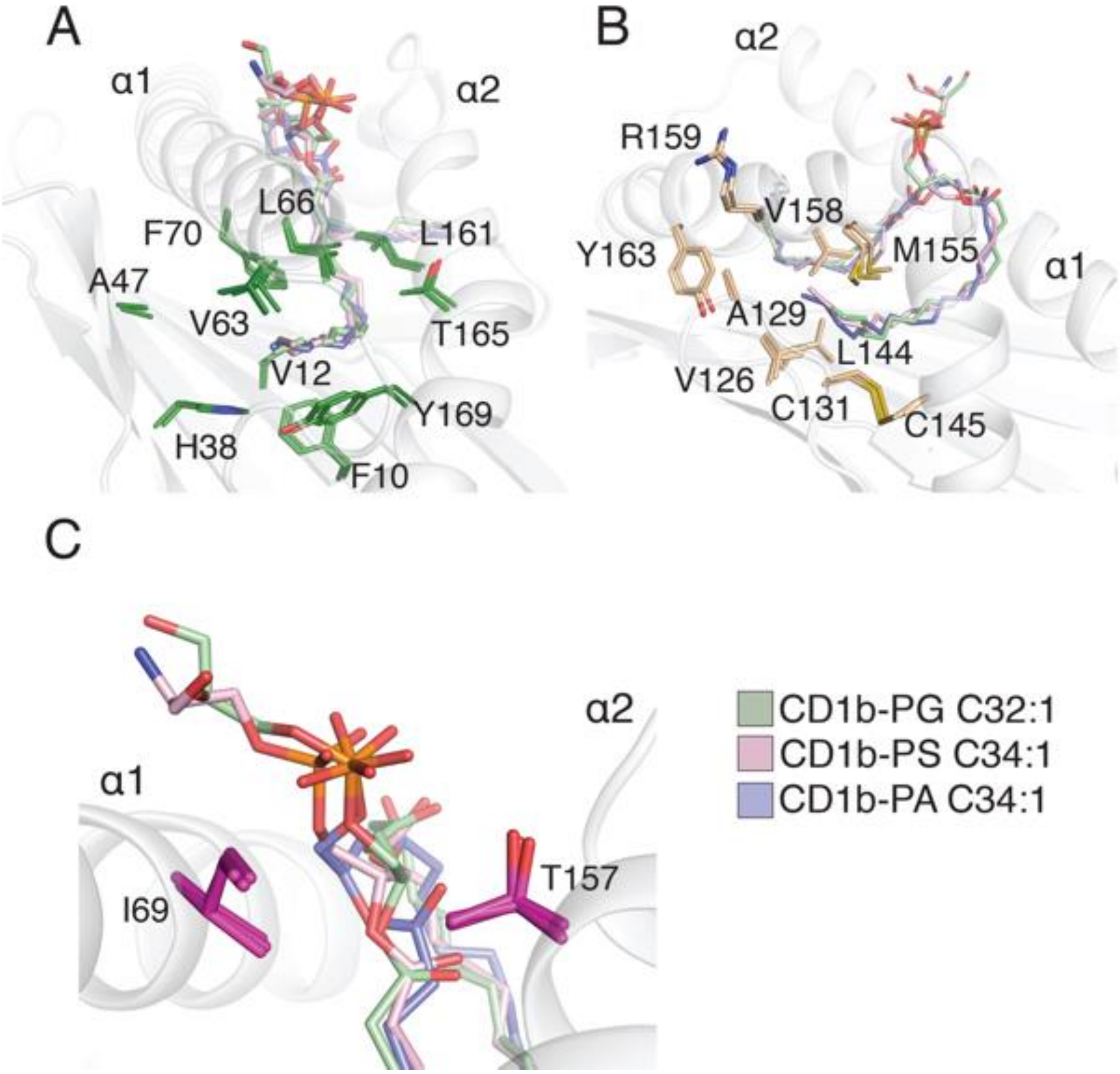

Fig. S9. Phospholipid anchoring residues within CD1b pockets. Architecture overview of the (**A**) A´ and (**B**) C´-grooves, as well as (**C**) the overlay of phospholipid headgroup antigens and contact residues. Stick representation of each residue comprising the (**A**) A´ (green) and (**B**) C´ (light brown) grooves from each crystal structure are overlayed and represented as sticks. (**C**) Residues making contacts with the phospholipid antigen headgroup and glycerol moiety are coloured in magenta.

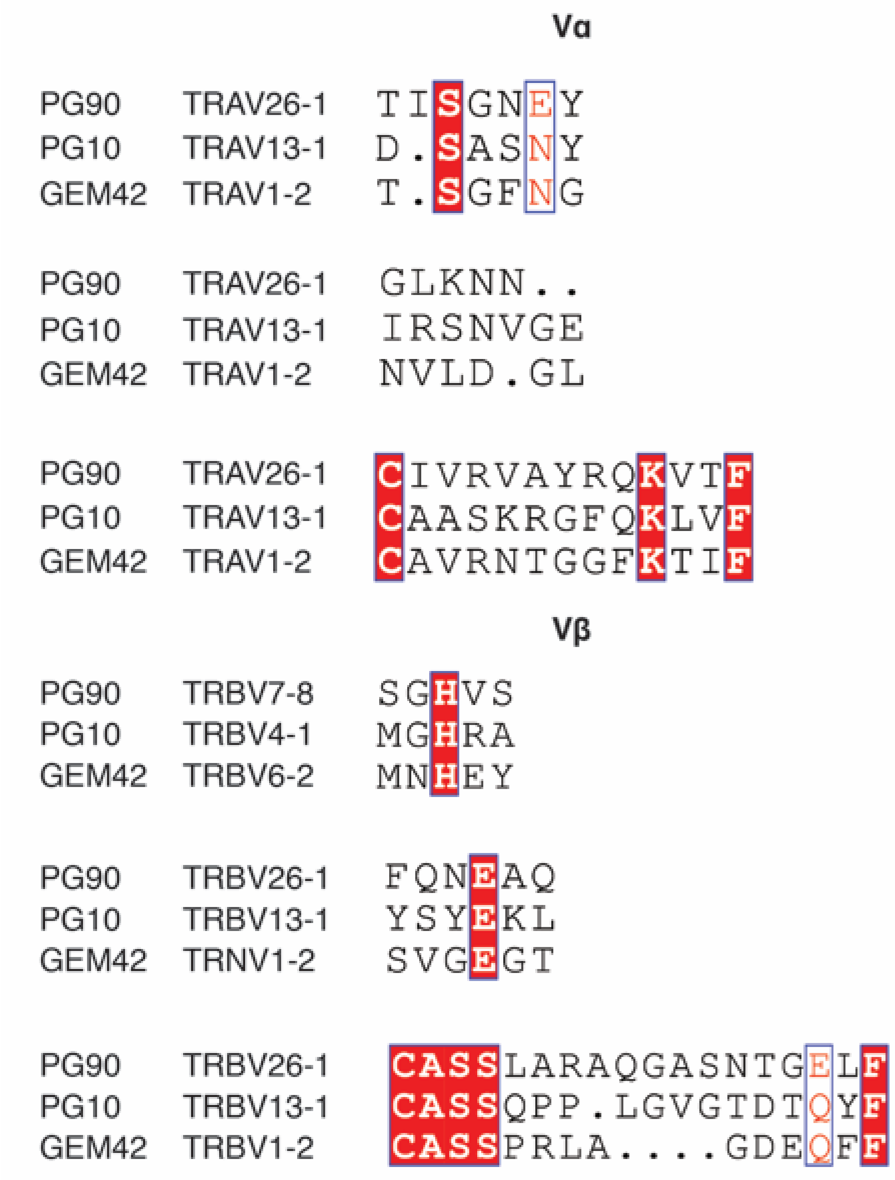

Fig. S10. Sequence alignments of TCR Vα and Vβ regions. Amino acid sequence alignments of the Vα and Vβ CDR loops of CD1b restricted PG90 TCR (TRAV26-1, TRBV7-8), PG10 TCR (TRAV13-1, TRBV4-1) and GEM42 TCR (TRAV1-2, TRBV6-2). Strict sequence-identical residues are represented with a red background in white text. Residues with similar side chain properties are visualised in white background with red text. Sequence similarities in groups are indicated by blue boxes. Sequence alignments were generated in Clustal Omega, and visualising using ESpript 3.0.

Table S1. Data collection and refinement statistics.

	CD1b-PG	CD1b-PA	CD1b-PS	PG90 TCR	PG90 TCR-CD1b-PG
**Space group**	$P2_12_12_1$	$P2_12_12_1$	$P2_12_12_1$	$P2_12_12_1$	C2
**Resolution range (Å)**	32.99 – 1.38 (1.40 – 1.38)	57.19 – 2.10 (2.16 – 2.10)	40.61 – 1.71 (1.74 – 1.71)	59.91 – 2.50 (2.60 – 2.50)	72.77 – 2.82 (2.97 – 2.82)
**Cell dimensions (Å, °)**	57.89, 80.28, 92.70	57.19, 79.53, 84.71	57.25, 78.55, 84.79	41.15, 128.08, 169.68	152.01, 82.97, 90.16, β=94.88
**Total No. of reflections**	1288604 (62278)	162492 (13192)	604027 (31931)	221289 (23373)	100887 (14897)
**No. of unique reflections**	87772 (4196)	22941 (1831)	42110 (2211)	32110 (3558)	27072 (3931)
**Multiplicity**	14.7(14.8)	7.1 (7.2)	14.3 (14.4)	6.9 (6.6)	3.7 (3.8)
**Completeness (%)**	98.4 (96.8)	100.0 (100.0)	100.0 (100.0)	100.0 (100.0)	99.8 (100.0)
**CC (1/2)**	0.999 (0.623)	0.985 (0.638)	0.999 (0.588)	0.982 (0.607)	0.996 (0.749)
**R_{pim} (%) [a]**	3.5 (55.9)	15.1 (55.9)	5.4 (60.1)	12.9 (56.7)	12.9 (58.5)
**Mean I/ $\sigma(I)$ [b]**	17.1 (2.0)	7.6 (2.0)	14.4 (2.0)	6.8 (2.1)	5.3 (2.0)
**R_{factor}/R_{free} (%) [c]**	18.54/20.35	18.35/23.13	19.31/22.99	21.10/24.20	22.00/25.90
**Non-hydrogen atoms**	4011	3501	3570	7280	6611
**Macromolecules**	3065	3022	3033	6912	6364
**Ligands**	289	203	202	2	118
**Water**	647	276	335	366	129
**Protein residues**	378	376	377	880	809
**R.M.S.D. from ideality**					
**Bond Lengths (Å)**	0.01	0.01	0.01	0.11	0.11
**Bond Angles (°)**	1.08	1.09	1.05	1.12	1.62
**Ramachandran Plot**					
**Favoured Region (%)**	99.74	98.9	99.21	96.39	96.73
**Allowed Region (%)**	0.26	1.1	0.79	3.38	3.14
**Outliers (%)**	0	0	0	0.23	0.13
**B-factors (Å^2)**					
**Average B-factors**	23.43	22.11	26.49	37.9	53.18
**Average Macromolecule**	19.84	2073	24.95	38.23	53.32
**Average Ligand**	33.92	34	34.85	52.02	58
**Average Water**	35.58	28.52	35.45	31.61	41.54
**PDB code accession**	5WKI	5WKG	5WKE	5WJO	5ML1

[a] $R_{pim} = \sum_{hkl} [1/(N - 1)]^{1/2} \sum_i | I_{hkl,\ i} - \langle I_{hkl} \rangle | / \sum_{hkl} \langle I_{hkl} \rangle$, [b] $\sigma(I)$ is the estimated standard deviation of the integrated intensity (I). **c:** $R_{factor} = \sum_{hkl}||F_o| - |F_c|| / \sum_{hkl}|F_o|$ for all data except 5%, which were used for R_{free} calculation. Highest resolution shell is shown in parenthesis.

Table S2. Contacts between PG90 TCR and CD1b-PG.

**TCR Gene**	**TCR residue**	**CD1b**	**Bond Type**
CDR1α	Gly29	Glu156	VDW
CDR1α	Asn30-Oδ1	Glu156, Thr157-N-Oγ1, Ile160	VDW, HB
CDR1α	Tyr32	Gly153	VDW
CDR2α	Leu51	Gln152	VDW
CDR2α	Asn53-Oδ1	Gln152-Cδ-Nε2-Oε1	VDW, HB
CDR3α	Ala93	Thr157	VDW
CDR3α	Tyr94-OH	Glu65-Oε2	HB
CDR3α	Tyr94	Glu65, Ile69, Val72	VDW
CDR3α	Arg95-NH2-Nε	Glu65-Oε1, Oε2	SB, HB
CDR3α	Arg95	Glu65, Glu68	VDW
CDR1β	Val30	Arg79	VDW
CDR2β	Gln50	Phe75	VDW
CDR3β	Leu96-O	Arg79-NH1-NH2	HB, VDW
CDR3β	Ala97-O	Arg79-NH1	HB
CDR3β	Arg98-Nε-NH1-NH2	Glu80-Oε1-Oε2, Asp83-Oδ1-Oδ2	HB, SB
CDR3β	Arg98	Arg79, Glu80, Asp83, Phe84	VDW
CDR3β	Ala99-N	Glu80-Oε2, Tyr151, Ile154	HB, VDW
CDR3β	Gln100	Tyr151	VDW
CDR3β	Gly101	Gln150, Tyr151	VDW
**TCR Gene**	**TCR residue**	**PGV**	**Bond Type**
CDR1α	Tyr32-OH	O4	HB, VDW
CDR3α	Arg91	C19, O5, C20	VDW
CDR3α	Ala93	C19, O6, C20, O2, O5	HB, VDW
CDR3α	Tyr94	O6, O9, C16,	VDW
CDR3α	Gln96-Nε2	C20, O6, O7, C21	HB, VDW
CDR3β	Ala97-O	O7, C21	HB, VDW
CDR3β	Arg98	C21	VDW
CDR3β	Ala99	O3	VDW
CDR3β	Gln100	C19, O5, O4	VDW
CDR3β	Gln100-N-O	O3, O5, P	HB, VDW

VDW: Van der Waals Interaction (cut-off of 4 Å), HB: Hydrogen Bond (cut-off of 3.5Å), SB: Salt Bridge (cut-off of 4.5 Å).
